# A Retrospective Analysis of the Diagnostic Accuracy in Diagnosing OPMD and Benign Oral Lesions

**DOI:** 10.1111/odi.70253

**Published:** 2026-02-18

**Authors:** Griselda Pedroso Acosta, Lucio Torelli, Theodora Magdalena Bogdan Preda, Roberto Di Lenarda, Matteo Biasotto, Katia Rupel, Giulia Ottaviani

**Affiliations:** ^1^ Department of Surgical, Medical and Health Sciences University of Trieste Trieste Italy; ^2^ Maxillofacial Surgery and Odontostomatology Clinic, Maggiore Hospital Azienda Sanitaria Universitaria Giuliano Isontina Trieste Italy

**Keywords:** clinical diagnosis, diagnostic concordance, early detection, histological diagnosis, oral potentially malignant disorders

## Abstract

**Objective:**

The clinical suspicion of a neoplastic lesion necessitates prompt diagnostic assessment through oral biopsy. However, the failure to accurately recognize an oral potentially malignant disorder (OPMD) or the misdiagnosis of a benign lesion can lead to diagnostic delays with significant impact on the patient's health. This study aimed to evaluate the diagnostic accuracy of clinical diagnosis compared to the histopathological diagnosis, focusing on OPMDs and suspected benign lesions in an Italian university hospital.

**Methods:**

A retrospective analysis was conducted on patients with a clinical diagnosis of benign lesions or OPMDs at the Oral Medicine and Pathology Unit (Maggiore Hospital, Trieste, Italy). Expert clinicians performed the clinical assessment, followed by biopsy and histopathological analysis. Diagnostic accuracy was evaluated through sensitivity, specificity, positive predictive value, and negative predictive value. Cohen's kappa (*κ*) coefficient was used to determine concordance between clinical and histological diagnoses.

**Results:**

The histopathological analysis diagnosed the presence of benign lesions in 74.7% of cases and OPMDs in 25.3%. Sensitivity was highest for odontogenic cysts and tumors (92.06%) and lowest for ulcerative lesions/TUGSE (37.50%). Specificity exceeded 95% across most considered categories. The overall concordance between clinical and histological diagnoses was substantial (*κ* = 0.70).

**Conclusion:**

The misdiagnosis of OPMDs or benign lesions leads to diagnostic delays which seriously impact on the patient's therapeutic process. This survey highlights both high diagnostic accuracy and strong concordance between clinical and histological diagnoses. Integrating advanced diagnostic tools and constant clinicians' training is essential to minimize misdiagnoses.

## 1 Introduction

The oral cavity is a complex anatomical region that mirrors systemic health and could exhibit a variety of lesions, including oral potentially malignant disorders (OPMDs). Globally, benign lesions including, but not limited, to fibromas and odontogenic tumors are common and often linked to trauma, infections, or congenital anomalies (Kumari et al. 2022). In contrast, OPMDs—following the latest classification established during the WHO Collaborating Centre Workshop on OPMDs held in Glasgow in 2020—include leukoplakia, proliferative verrucous leukoplakia, erythroplakia, oral submucous fibrosis, oral lichen planus, actinic keratosis (actinic cheilitis), palatal lesions in reverse smokers, discoid lupus erythematosus, dyskeratosis congenita, oral lichenoid lesions, and oral graft‐versus‐host disease (GVHD) (Warnakulasuriya et al. 2021). For clinicians, OPMDs represent a critical field due to their associated risk of malignant transformation. According to Kumari et al. (2022), OPMDs affect approximately 4.47% of the population, with malignant transformation rates between 14% and 50% for erythroplakia and up to 16% for nonhomogeneous leukoplakia (Warnakulasuriya and Kujan 2021). Compared to unaffected oral mucosa, OPMDs exhibit a significantly higher risk of cancer development, reinforcing the importance of their early identification. Therefore, early detection is crucial, as highlighted by the WHO/IARC GLOBOCAN data (American Cancer Society 2023), which reports a 5‐year survival rate for oral squamous cell carcinoma (OSCC) around 94% for localized disease, 63% for regional spread, and 38% for distant metastases.

Indeed, clinical evaluation under white light remains a noninvasive, accessible diagnostic tool; however, its sensitivity and specificity for OPMDs vary widely (50%–75%), particularly depending on clinician expertise (González‐Moles et al. 2022). González‐Moles et al. (2022) highlight the challenges physicians face in accurately diagnosing OPMDs, particularly when nuanced clinical presentations overlap with benign/reactive lesions, leading to potential underdiagnosis or misclassification. Diagnostic errors, especially in early‐stage lesions, may result in high‐risk patients being excluded from necessary follow‐up programs, delaying intervention and increasing the risk of disease progression. Actually, Maloney et al. (2024) stressed the need for vigilant monitoring of nonhomogeneous leukoplakia, given its higher malignant potential compared to the homogeneous variant. These data underline the importance of a focused and accurate preliminary clinical evaluation, which impacts on the need and timing to proceed with biopsy sampling. Therefore, histological examination remains the gold standard for confirming or ruling out clinical diagnosis. Rich et al. (2023) reinforced the concept that the clinical evaluation alone is insufficient for a definitive diagnosis, underlining the indispensable role of histology in validating clinical impressions and guiding appropriate treatment strategies.

The clinical suspicion of a neoplastic lesion warrants immediate diagnostic assessment through oral biopsy. However, the failure to accurately recognize an OPMD or the misdiagnosis of a benign lesion may result in diagnostic delays, potentially leading to significant consequences for the patient. Therefore, this retrospective study aims to assess the diagnostic accuracy of clinical suspicion, compared to the definitive diagnosis obtained through oral biopsy, including cases of suspected OPMDs and benign lesions in an Italian university hospital.

## 2 Materials and Methods

### 2.1 Study Population and Data Extraction

A retrospective observational study was conducted on a total of 1179 patients referred at the Oral Medicine and Pathology Unit (Maxillofacial Surgery and Odontostomatology Clinic, Maggiore Hospital, Trieste, Italy) between January 2019 and December 2024. The study protocol was approved by the institutional Ethics Committee (n. 2/2024) in accordance with the Helsinki Declaration.

The data were extracted as follows: age, demographic characteristics, past and present medical histories, lesion localization along with objective examination, histological diagnosis. To ensure diagnostic consistency and minimize interobserver variability, all clinical evaluations were performed by three senior staff clinicians, each possessing a minimum of 5 years of specific practice in oral medicine within our university hospital setting. Throughout the study, any ambiguous or borderline cases were discussed collectively until a consensus was reached, ensuring a high level of diagnostic reliability. Only lesions clinically diagnosed as benign or OPMDs and subsequently confirmed by histopathological examination were included in the analysis.

One of the Authors (GPA) was responsible for data extraction from the database and, together with another Author (GO), data were checked for completeness and accuracy. Data excluded from the study were based on the following criteria: (i) incomplete clinical records; (ii) absence of a confirmatory histological diagnosis; (iii) histopathological reports deemed inconclusive or ambiguous by the reviewing pathologists. For all included cases, the final diagnosis was systematically confirmed through histopathological examination, ensuring the diagnostic accuracy of the study cohort.

To ensure methodological rigor and standardization, clinical and histological diagnoses were allocated into macrocategories—OPMDs and benign tumors—adopting the WHO classification that provides an internationally recognized framework for head and neck tumors, ensuring consistency and accuracy in categorizing lesions, as outlined by El‐Naggar et al. (2017). The alignment with WHO classification, an internationally recognized diagnostic categorization, strengthens the reliability of the analysis and facilitates meaningful comparisons with literature, thus providing consistent and reproducible data.

Additionally, lesions' diagnoses were further classified into subcategories according to Neville's Oral and Maxillofacial Pathology textbook (5th edition) (Neville et al. 2023). This detailed categorization included odontogenic cysts and tumors, foreign body/granulation tissue, periodontal disease, allergic and immunological diseases, oral manifestations of dermatological diseases, salivary gland disorders, epithelial pathologies, bone pathologies, soft tissue tumors, and ulcer/TUGSE. For odontogenic lesions specifically, we adopted the classification system from Soluk‐Tekkesin and Wright (2018), summarizing the changes introduced in the 4th edition of the WHO classification. This approach allowed for a precise differentiation between various odontogenic cysts and tumors, reinforcing the diagnostic accuracy of the study.

### 2.2 Statistical Analysis

The reference standard for oral lesions diagnosis was the histological analysis.

Descriptive statistics summarized age and demographic characteristics. Diagnostic accuracy between clinical and histological diagnoses was evaluated through sensitivity, specificity, positive predictive value (PPV), and negative predictive value (NPV), with 95% confidence intervals (CIs). Concordance was assessed using the Kappa coefficient (*κ*), which ranges from zero for no agreement to 1 for perfect agreement, and the following standards for strength of agreement for the kappa coefficient have been proposed: 0.01–0.20, slight; 0.21–0.40, fair; 0.41–0.60, moderate; 0.61–0.80, substantial; and > 0.80 almost perfect (Sindi and Aljohani 2024). The following analyses were performed for the whole sample.

SPSS software 16.0 (SPSS Inc., Chicago, Illinois, USA) and MedCalc software 12.3.3.0 (MedCalc Software, Mariakerke, Belgium) were used to perform the statistical analyses. A *p* value < 0.05 was used for rejection of the null hypothesis.

## 3 Results

### 3.1 Demographic Data

A total of 1179 medical records were retrieved, which constituted the whole sample. Among all of these cases, a total of 831 presented with a full set of the recorded data and constituted the fully classified sample for further analyses. Out of the total sample, 337 patients (40.5%) were male and 494 (59.5%) were female, with a mean age of 65 years (range: 12–100 years; SD: 17.72 years). The highest number of cases involved patients in their 60s and 70s (43.9%), whereas only 1.9% were younger than 20 years. Table 1 details the distribution of cases by age and gender.

**TABLE 1:** Distribution of cases by age and gender (*n* = 831).

Age group	Total cases *N* (%)	Male cases *N* (%)	Female cases *N* (%)
< 20	16 (1.9)	9 (2.7)	7 (1.4)
20–39	82 (9.9)	43 (12.8)	39 (7.9)
40–59	226 (27.2)	100 (29.6)	126 (25.5)
60–79	365 (43.9)	139 (41.3)	226 (45.8)
80–89	123 (14.8)	43 (12.7)	80 (16.2)
90+	19 (2.3)	3 (0.9)	16 (3.2)
Total	831 (100)	337 (40.5)	494 (59.5)

Table 2 summarizes the distribution of lesions according to anatomical sites. The gingiva was the most commonly affected site (22.0%), followed by the lining mucosa (19.5%). Less frequent sites included the labial commissure (1.3%) followed by soft palate (2.3%).

**TABLE 2:** Distribution of lesions by anatomical site (*n* = 831).

Anatomical site	*N* (%)
Labial commissure	11 (1.3)
Alveolar ridge	82 (9.9)
Dorsal surface of tongue	45 (5.4)
Lower lip	110 (13.2)
Upper lip	26 (3.1)
Lateral surfaces of tongue	60 (7.2)
Gingiva	183 (22.0)
Lining mucosa	162 (19.5)
Hard palate	51 (6.1)
Soft palate	19 (2.3)
Floor of the mouth	33 (4.1)
Ventral surface of tongue	21 (2.5)
Retromolar area	28 (3.4)

According to the 2017 WHO classification (El‐Naggar et al. 2017), lesions were divided between OPMDs and benign tumors. The distribution of clinical and histological diagnoses across these macrocategories is summarized in Table 3. Overall, benign tumors represented 78.8% of clinical diagnoses and 74.7% of histological diagnoses. The clinical suspect of OPMD was posed for 21.2% of evaluated lesions and histologically confirmed in 25.3% of cases. The Kappa coefficient (*κ* = 0.70) indicated a good level of agreement between the diagnoses with an observed concordance rate of 90.13%.

**TABLE 3:** Frequency of macrocategory distribution according to the clinical and histological diagnoses.

Macrocategories	Clinical diagnoses *N* (%)	Histological diagnoses *N* (%)
OPMD	176 (21.2)	210 (25.3)
Bening tumors	655 (78.8)	621 (74.7)

*Note:* Data are expressed as percentage distribution of OPMDs and benign tumors. OPMD, oral potentially malignant disorder.

Detailing the clinical diagnosis, cases were categorized into specific subcategories according to Neville's classification published in the Oral and Maxillofacial Pathology textbook (Neville et al. 2023) and the classification system from Soluk‐Tekkesin and Wright (2018) for odontogenic lesions. Table 4 resumes the diagnostic accuracy of clinical evaluation compared to histological diagnosis assessed across the diseases' subcategories. The results highlighted a high level of diagnostic accuracy across most subcategories, with high rates for specificity and NPV. Odontogenic cysts and tumors demonstrated excellent identification, with 99.87% specificity and 99.35% NPV. Similarly, bone pathologies and ulcer/TUGSE showed nearly perfect specificity (99.50% and 100.00%, respectively) and high NPV (99.75% and 98.18%, respectively). Other subcategories, including oral manifestations of dermatological diseases, salivary gland disorders, and soft tissue tumors, reported a consistent specificity above 96% and NPV values exceeding 93%. While sensitivity and PPV varied, these results suggest that clinical diagnoses are generally predictable when histological analysis is still pending. Overall, these data reflect a strong concordance between clinical and histological diagnoses, thus reinforcing the reliability of clinical evaluations by expert clinicians for most oral of the analyzed categories (90.13%).

**TABLE 4:** Diagnostic accuracy parameters for each subcategory considered.

Subcategory	Parameter	Mean (95% CI)
Odontogenic cysts and tumors	Sensitivity	92.06 (82.44–97.37)
	Specificity	99.87 (99.28–100.00)
	PPV	98.31 (89.09–99.76)
	NPV	99.35 (98.51–99.72)
Foreign body/granulation tissue	Sensitivity	62.50 (35.43–84.80)
	Specificity	98.53 (97.44–99.24)
	PPV	45.45 (29.73–62.14)
	NPV	99.26 (98.61–99.60)
Periodontal disease	Sensitivity	71.43 (29.04–96.33)
	Specificity	98.79 (97.78–99.42)
	PPV	33.33 (18.74–52.02)
	NPV	99.75 (99.21–99.92)
Allergic and immunological diseases	Sensitivity	100.00 (59.04–100.00)
	Specificity	99.26 (98.39–99.73)
	PPV	53.85 (34.46–72.14)
	NPV	100.00 (99.54–100.00)
Oral manifestations of dermatological diseases	Sensitivity	79.39 (71.45–85.96)
	Specificity	97.86 (96.49–98.80)
	PPV	87.39 (80.66–92.02)
	NPV	96.21 (94.77–97.26)
Salivary gland disorders	Sensitivity	82.26 (70.47–90.80)
	Specificity	99.09 (98.13–99.63)
	PPV	87.93 (77.55–93.89)
	NPV	98.58 (97.59–99.16)
Epithelial pathologies	Sensitivity	80.93 (75.33–85.74)
	Specificity	91.60 (89.07–93.70)
	PPV	79.25 (74.42–83.38)
	NPV	92.37 (90.29–94.04)
Bone pathologies	Sensitivity	91.67 (73.00–98.97)
	Specificity	99.50 (98.74–99.86)
	PPV	84.62 (67.26–93.64)
	NPV	99.75 (99.07–99.93)
Soft tissue tumors	Sensitivity	85.13 (80.31–89.16)
	Specificity	96.44 (94.56–97.81)
	PPV	91.97 (88.13–94.64)
	NPV	93.13 (91.05–94.75)
Ulcer/TUGSE	Sensitivity	37.50 (18.80–59.41)
	Specificity	100.00 (99.54–100.00)
	PPV	100.00 (66.37–100.00)
	NPV	98.18 (97.53–98.65)

*Note:* Data are expressed as percentages.

Abbreviations: CI, confidence interval; NPV, negative predictive value; PPV, positive predictive value.

## 4 Discussion

Accurate diagnosis of an OPMD is essential for effective patient management and timely intervention when indicated. However, the clinical identification of OPMDs remains challenging due to their heterogeneous presentation and frequent overlap with benign or reactive lesions. In this survey, we focused on lesions clinically diagnosed as OPMDs or benign, based on the assumption that lesions exhibiting unequivocal malignant features are usually promptly biopsied and therefore associated with a minimal margin of diagnostic uncertainty. In contrast, lesions with ambiguous or nonspecific clinical features represent the main source of diagnostic difficulty in daily practice.

The present study evaluated the concordance between clinical assessment and histopathological diagnosis across 831 oral biopsies performed in a university‐based Oral Medicine and Pathology Unit, providing a real‐world overview of diagnostic performance in routine clinical activity.

Our study highlights a significant concordance between clinical and histological diagnoses, as reflected by a substantial Kappa agreement (*κ* = 0.70) in line with previous reports (Rich et al. 2023; Sindi and Aljohani 2024), supporting the reliability of clinical evaluation when performed by experienced clinicians. By employing standardized classifications—specifically, the 4th edition of the WHO classification for head and neck tumors (El‐Naggar et al. 2017) and Neville's classification (5th edition) (Neville et al. 2023)—we ensured that diagnostic criteria adhered to internationally recognized guidelines. This methodological consistency strengthens the robustness of our findings and allows meaningful comparison with previously published data, including concordance rates reported by Rich et al. (2023) and González‐Moles et al. (2022).

The demographic distribution of the present survey showed a higher prevalence of oral lesions among women (59.5%) compared to men (40.5%), consistent with findings from Golnoush et al. (2022) and Radwan‐Oczko et al. (2022). This difference is likely related to health‐seeking behavior rather than true biological disparities, as women may be more prone to seek clinical evaluation. The gingiva (22.0%) and lining mucosa (19.5%) were the most frequently involved anatomical sites, in line with previous studies identifying these regions as particularly susceptible to chronic irritation and reactive changes (Fattahi et al. 2014; Ramos et al. 2021). The labial commissure (1.3%) and soft palate (2.3%) were the least involved sites, as previously reported by Shamim et al. (2008).

With regard to diagnostic accuracy across lesion subcategories, high specificity and negative predictive values were observed for most categories, indicating that clinically negative assessments were generally reliable (Boza‐Oreamuno and López‐Soto 2020). Odontogenic cysts and tumors showed particularly high diagnostic accuracy, likely due to their distinctive clinical and radiographic features, as also reported by Neville et al. (2023). In contrast, sensitivity was markedly lower for ulcer/TUGSE and reactive lesions, confirming that these entities represent a major diagnostic challenge. The low sensitivity observed for ulcer/TUGSE (37.5%) is consistent with previous reports (González‐Moles et al. 2022; Setti et al. 2025; Benitez et al. 2019) and can be explained by their nonspecific clinical presentation. Ulcer/TUGSE lesions often appear as persistent ulcers and may clinically overlap with a wide spectrum of traumatic, inflammatory, infectious, or neoplastic conditions, making accurate clinical classification particularly challenging during the clinical evaluation (Setti et al. 2025; Benitez et al. 2019). This overlap reinforces the need for histopathological confirmation, especially in persistent ulcerative lesions. Similar diagnostic difficulties have been reported for other benign and reactive lesions, including fibromas, papillomas, cysts, gingival hyperplasia, reactive leukoplakia, angular cheilitis, and traumatic ulcers (Rich et al. 2023; Purushothaman et al. 2023).

The low positive predictive value observed for foreign body/granulation tissue (45.45%) and periodontal disease (33.33%) further highlights the risk of false positive clinical impressions when inflammatory or reactive processes mimic dysplastic changes. Such overlap complicates the diagnostic process and supports the routine use of biopsy to avoid misclassification. While diagnostic accuracy varied across subcategories, odontogenic cysts and tumors consistently demonstrated high sensitivity, specificity, PPV, and NPV, reflecting their more recognizable clinical features.

Overall, the major diagnostic challenge does not lie in the identification of clearly benign lesions, but rather in the correct clinical stratification of lesions with ambiguous or overlapping features, particularly within the spectrum of OPMDs. While experienced clinical evaluation represents an essential first step, histological examination remains the cornerstone for definitive diagnosis. Adjunctive diagnostic tools, including molecular biomarkers and imaging techniques such as narrow‐band imaging, may further improve diagnostic sensitivity in selected cases, but should be considered complementary to clinical judgment rather than substitutes (Carreras‐Torras and Gay‐Escoda 2015; Ottaviani et al. 2016; Zhang et al. 2023).

A potential limitation of this study is the selection bias inherent in the exclusion of 348 cases from the initial cohort due to incomplete clinical records or ambiguous histopathological data. While this stringent selection was necessary to ensure data integrity and to base our findings exclusively on cases with a confirmed gold standard diagnosis, we acknowledge that it may limit the generalizability of our results. Moreover, the retrospective design and single‐center setting may further limit the generalizability of the findings to different populations or clinical contexts. Nevertheless, the large sample size, standardized diagnostic criteria, and consistent clinical environment strengthen the reliability of the present observations.

## 5 Conclusions

Clinical evaluation represents a crucial first step in the management of oral lesions, but it cannot replace histopathological confirmation, particularly when dealing with OPMDs or lesions with unspecific clinical features. The present study demonstrates a substantial concordance between clinical and histological diagnoses across a wide spectrum of oral lesions, supporting the reliability of clinical assessment performed by experienced clinicians. However, reduced sensitivity in specific subcategories highlights the persistent risk of misclassification and diagnostic delay when relying on clinical examination alone. Integrating adjunctive diagnostic tools and promoting continuous clinician training may further improve early detection and contribute to better patient outcomes.

## Author Contributions

**Griselda Pedroso Acosta:** formal analysis, investigation, writing – original draft. **Lucio Torelli:** data curation, formal analysis, methodology. **Theodora Magdalena Bogdan Preda:** conceptualization, investigation, methodology. **Roberto Di Lenarda:** investigation, validation. **Matteo Biasotto:** investigation, validation. **Katia Rupel:** investigation, validation. **Giulia Ottaviani:** conceptualization, methodology, supervision.

## Conflicts of Interest

The authors declare no conflicts of interest.

## Data Availability

The data that support the findings of this study are available on request from the corresponding author. The data are not publicly available due to privacy or ethical restrictions.
